# Curantur or Curentur

**Published:** 1884-09

**Authors:** Ad. Lippe


### CURANTUR OR CURENTUR.

Ad. Lippe, M. D.

Dr. Dudgeon comes out in the July number of The Homœopathic Physician, and espouses the cause of Dr. Hughes, and with him contends that the complete homœopathic formula should always read *curentur;* that *curantur* would be a blunder because it would show that our formula (with *curantur* accepted) was an acknowledgment of "a law," while *curentur* would mean that we had only to do with a rule, good at times, but never to be accepted or applied imperatively. The plain fact is, that a question of vital importance has been asked and this question is: Did Hahnemann establish an exclusive system of therapeutics, or did he advocate eclecticism and call it Homœopathy? Did Hahnemann call Homœopathy a healing art, based on an immutable law expressed under the formula *similia similibus curantur?* Has not this law become obnoxious to men who, professedly homœopaths, in reality adhere to and advocate eclecticism, and to shield themselves from censure and exposure boldly claim that Hahnemann advocated their eclecticism. The formula, *similia similibus curantur*, has been the accepted formula of all medical men who profess to practice Homœopathy *till* a dose of Ruta graveolens was administered to Dr. Richard Hughes through the medium of The Homœopathic Physician, and on a sudden the dimness of sight for which Dr. R. Hughes had discovered Ruta to be a specific vanished, and it appeared before his sharpened sight that the finding of specifics for specific diseases and the proposed incorporation into our materia medica pura of pathological notions had been detected and that *his* game was likely to be balked, and therefore he resorted to more heroic means to extinguish Homœopathy and replace it by vile eclecticism. On a sudden this learned man detected that the perpetuity of pure Homœopathy was secured as long as the old formula prevailed, and, following in the footsteps of the *Homœopathic Times*, his aim was to make it appear that Hahnemann gave us not an immutable law, but a good rule, which could be applied if the individual judgment of the healer thought proper to do so, or it could be set aside at will. Hahnemann clearly shows in paragraphs 53–56 that there can be but one sole aid-bringing law in therapeutics. He advocates an exclusive law, and that law the homœopathic law of cure.

The most remarkable fact is that the *British Journal of Homœopathy*, the organ of Drs. Hughes, Dudgeon & Co., still shows upon its paper cover the old and well-known formula, *similia similibus curantur*. The question that at once occurs is, If this rendering is wrong, how is it that they still permit it to occupy so conspicuous a place upon their journal? What do they mean by flaunting upon the outside of this periodical a creed which within its pages they are engaged in vigorously denouncing? If it is an error, why have they not discovered one so obvious years ago? If they have the hardihood to display it upon their title-page, right over the very pages that expose their disbelief in it, what probability is there that they believe anything at all connected with the cause they profess to serve? Did they *ever* believe in it? Have they not always, as they evidently do now, pirate-like, sailed under false colors?

Certain it is that the pages of that journal have given increasing evidence from year to year that its editors used the formula only as a blind; and that their settled purpose was to instill into the minds of their readers a profound distrust of its truth. Now after the administration of a dose of Ruta one of the learned editors is enabled to discover that the formula upon the title-page (which formula he still flourishes in its old place) is all wrong!

History always repeats itself. In America, where we are in the habit of moving more rapidly than the people of the "old country," we had, a few years ago, an ably conducted journal known as the New York *Homœopathic Times*. By and by the exactions of a natural law of cure became an onerous burden to the freedom-seeking editor, whereupon he repudiated it, denying its existence, and waging war upon its followers.

The *British Journal of Homœopathy* has caught the same epidemic disease and exhibits the same symptoms. Perhaps Professor Pasteur may be able to find the cause of the disease, and to raise a graft of it in his "nursery" with which to inoculate the learned editors and cure them. If that fail, perhaps they may apply to the *other* wing of the homœopathic school and take a potentization of the product of the disease—say Dr. Hughes' or Dr. Dudgeon's papers raised to the CMM potency in a patent bottle-washing machine. If *this* sovereign specific fail to cure them, then their fate is inevitable. Like the *Times*, they will be obliged to drop the formula, and even the name, and thus, like the *Times*, become honest.

In conclusion, let us hope that the next number of the *British Journal of Homœopathy* will show its conversion to honesty by showing *curentur* upon its cover. Should it do so, we will have something to say on that subject. But if *curantur* remains, as of old, upon the cover, the editors will thereby confess themselves beaten badly—and that's all there is about it.

